# An unusual foreign body in the Cricopharynx; first case report Managed endoscopically

**Published:** 2013-03-25

**Authors:** A Kaur, A Singh, R Singal, M Singh, S Gupta

**Affiliations:** * Dept of Physiology, Maharishi Markandeshwer Institute of Medical Sciences and Research, Mullana, Ambala, India; ** Department Of Otolaryngology (E.N.T), Maharishi Markandeshwer Institute of Medical Sciences and Research, Mullana, Ambala, India; *** Dept of Surgery, Maharishi Markandeshwer Institute of Medical Sciences and Research, Mullana, Ambala, India; **** Dept of Radiodiagnosis and Imaging, Maharishi Markandeshwer Institute of Medical Sciences and Research, Mullana, Ambala, India

**Keywords:** Ayurvedic tablet, cricopharynx, dysphagia, hypopharyngoscopy, mercury

## Abstract

A foreign body at the cricopharynx level is a common problem to any otolaryngologist worldwide. Coins, pencil tips, screws are usually found in children but are rarely seen in adults in the cricopharynx. We present an unusual case in a 30-year-old female who swallowed an Ayurvedic tablet. She complained of dysphagia and was unable to swallow even liquids. To our surprise, on the X-ray of the neck, a radiopaque shadow was noted in the cricopharynx. We removed it by hypopharyngoscopy and in the follow up period, the patient had no squeals. Any foreign bodies in the cricopharynx should be removed quickly to avoid complications like erosion. To our knowledge, the radiopaque shadow of a tablet observed on the X-ray of the neck is the first case being reported in the world literature.

## Introduction

Swallowing is initiated by the voluntary action of collecting oral contents on the tongue and propelling them backward into the pharynx [**1**]. Foreign bodies tend to lodge in sites of constriction in the oesophagus. These are usually the points of natural narrowing at 15 and 25 centimetres, but the reason for impaction at these levels may have more to do with motility patterns than anatomy [**2**]. The space located in between the pharynx and the cervical oesophagus is a high-pressure zone known as the Upper oesophageal sphincter (UES). The physiological role of this sphincter is to protect against reflux of food into the airways as well as prevent entry of air into the digestive tract [**3**]. In our case, the Ayurvedic pill was successfully removed from the cricopharynx by rigid hypopharyngoscopy. If not managed on time, erosion and oedema can lead to dysphagia/obstruction, which may increase the morbidity and mortality.

## Case report

A 30-year-old female came to the emergency department of our institute with difficulty in swallowing since one day. The patient was not even able to have water. An assessment of the nervous system was made because neuromuscular disorders may present with dysphagia as an initial symptom. Proper history revealed that the patient was a known case of rheumatoid arthritis and was taking routine anti-inflammatory drugs. The prior day, the patient was advised to take ayurvedic tablets by relatives for immediate relief. The tablet was about I cm in diameter and was spherical in shape. The moment the patient took the first tablet with water, she started having discomfort in the throat. She took more water but was not relieved. She visited local doctors and was referred to our institute.

**Fig. 1:**
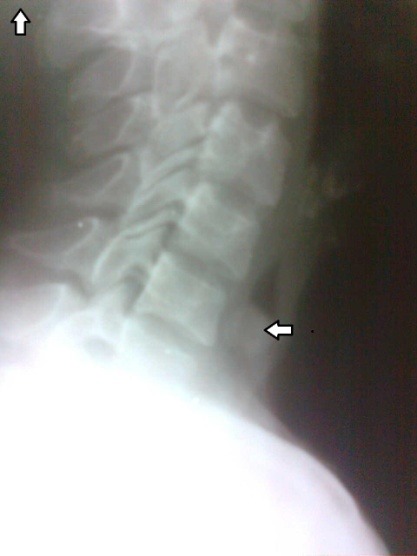
1Horizontal arrow shows the foreign body (tablet) in cricopharynx at C6-C7 level

 On arrival she had drooling of saliva; the oxygen saturation was between 70%-80% and the patient was not able to take anything orally since 12-14 hours. While performing an indirect laryngoscopy, we found pooling of saliva in both pyriform fossae. Taking history into account rigid oesophagoscopy under general anaesthesia was planned. All routine blood tests were in normal limits. The X-ray of the neck lateral view revealed a radiopaque spherical shadow at the lower level of cricopharynx, which was a surprise to us, as routinely tablets are not radiopaque. There was also a slight prevertebral widening in front of C6 and C7 (**Fig. 1**).

**Fig. 2:**
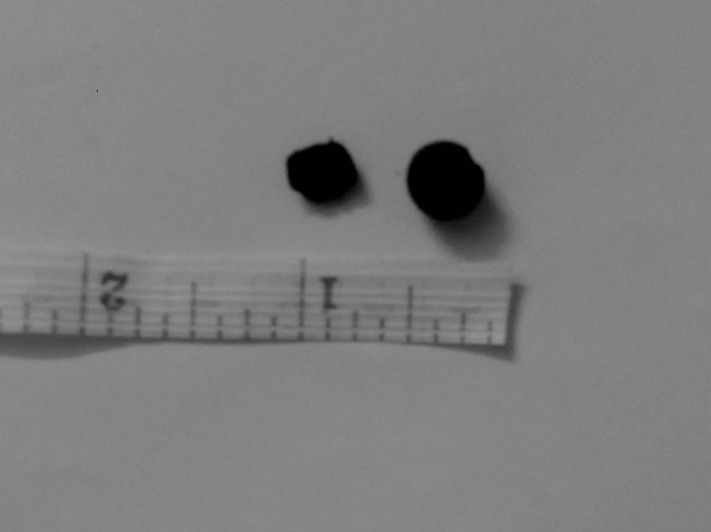
Tablet removed piecemeal

To understand what we were going to encounter, the patient’s husband was asked to provide another ayurvedic pill of the same kind. The tablet’s colour was black, it was spherical, stony hard and its approximate size was of 1 cm. Not knowing the contents of the pill, it was decided that it should be removed immediately because a long-standing tablet may cause erosion/obstruction of the wall of the oesophagus. The patient was shifted to operation theatre for rigid hypopharyngoscopy under general anaesthesia. On rigid hypopharyngoscopy, the tablet was visualised in lower border of the cricopharynx. The surrounding area was extremely congested and oedematous. With the help of a foreign body removal forceps, the tablet was grasped but some part of it broke, which made the removal action easier. The rest of the tablet affected the wall of the cricopharynx and was eventually removed (**Fig. 2**). On gross appearance, the pill’s colour was black; it was hard in consistency and of approximately 1.25 cm in size.

**Fig. 3:**
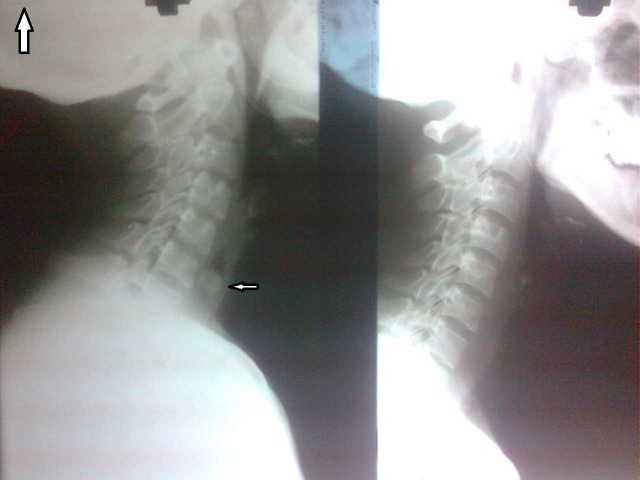
Postoperative radiograph showing normal cricopharynx

The postoperative X-ray of the neck confirmed the complete removal of the contents (**Fig. 3**).

 As there was an erosion of the wall of the oesophagus, the patient was kept nil orally along with routine intravenous antibiotics and steroids. After two days, liquids were started, followed by semisolids and a normal diet. The patient was discharged on the 4th day postoperatively, being totally relieved of symptoms.

## Discussion

A foreign body lodged in the cricopharynx can cause damage/obstruction to the airway. Children tend to ingest almost every type of objects which can cause complications [**4,5**]. The oesophageal foreign bodies are usually seen in children and some kind of adults, like prisoners or those who have oesophageal problems, mental retardation and psychiatric illness [**6**]. In this world, most of living persons would have ingested at least one foreign body in his lifetime either accidentally or intentionally [**7**]. 

 The visualisation of the pharynx/hypopharynx by indirect laryngoscopic examination may reveal foreign body or just pooling of saliva in the pyriform fossae. Pain while drinking (swallowing test) or the moving of trachea or larynx in a side-to-side motion (tracheal rock/laryngeal rub) suggest the presence of a foreign body [**8**]. Early endoscopic removal of the foreign body is necessary if stuck in the cricopharyngeal sphincter or oesophagus, by general anaesthesia. Hospital stay and morbidity can be decreased, only if treated as early as possible [**9**]. 

 Ayurveda medicine is a system of traditional medicine native in India and practiced in other parts of the world as an alternative medicine. Ayus means longevity and Veda is related to knowledge or science. According to a study, one out of every five ayurvedic herbal medicine products in south Asia contain potentially injurious contents as lead, mercury and arsenic [**10**]. Mercury is used in India by practitioners of alternative medicine, such as Ayurveda, siddha and unani medicine. Especially in Ayurveda, mercury is used in the form of bhasma that is obtained from incinerated mercury along with selected herbs.

In our case, the pill was stuck up in the cricopharynx; this might be of its large size and was causing difficulty in ingestion since one day. The shape and texture of the tablet (spherical, hard) was very challenging for management. Along with it, the patient was also suffering from rheumatoid arthritis so other connective disorders involving oesophagus and affecting its motility were also kept as a co-diagnosis. We managed the case successfully in the emergency without encountering any complications. In literature, abundant cases of foreign body cricopharynx of tablets along with blister pack have been reported, but a single ayurvedic tablet causing absolute dysphagia and the diagnosis of the tablet lodged in cricopharynx on X-ray is a rare clinical presentation.

 The present case is described to highlight the need for a proper history and examination in case of foreign body cricopharynx. In the case of a foreign body in the cricopharynx, the X-ray of the neck can be helpful and the surgeon should not wait for the tablet to dissolve by itself. Its immediate removal is necessary, as it may swell up due to its hygroscopic nature and may also cause erosion/oedema of wall of the oesophagus, depending on the nature of the constitutes.

## Conclusions

We recommend that the patients should take the tablets by cutting them in smaller bits before being swallowed or health education to the traditional healers on the risk associated with mercury or arsenic ingestion so that there will not be a future occurrence of the case. A single Ayurvedic tablet acting as a foreign body cricopharynx is a rare clinical presentation. X-rays are routine tests, but they should also be done in foreign body cases causing dysphagia as seen in our case. We managed the case on time and there was no further complication. 
